# Recurrent care proceedings and use of services for substance use disorder: A retrospective linked data cohort study of mothers in South London

**DOI:** 10.1111/add.70179

**Published:** 2025-08-27

**Authors:** Martha Canfield, Gail Gilchrist, Johnny Downs, Sam Norton

**Affiliations:** ^1^ Department of Psychology Glasgow Caledonian University Glasgow UK; ^2^ Department of Psychology, Health Psychology Section, Institute of Psychiatry, Psychology and Neuroscience King's College London London UK; ^3^ National Addiction Centre, Institute of Psychiatry, Psychology and Neuroscience King's College London London UK; ^4^ CAMHS Digital Lab, Department of Child and Adolescent Psychiatry, Institute of Psychiatry, Psychology and Neuroscience King's College London London UK; ^5^ Department of Inflammation Biology, Faculty of Life Sciences and Medicine King's College London London UK

**Keywords:** care proceedings, linked data, mothers, returning cases, substance use disorder, treatment

## Abstract

**Background and aims:**

In public family law cases (‘care proceedings’), many mothers return to proceedings after having a child removed. Substance use disorder (SUD) is a common feature in these cases. We used a linked dataset between SUD treatment services and family court to identify: i) the prevalence and estimated time for returning to care proceedings, ii) the characteristics of mothers who returned, and iii) differences in SUD treatment service use between mothers who returned to care proceedings and those who did not.

**Design:**

Retrospective study.

**Setting:**

South London and Maudsley NHS Mental Health Trust (SLaM) catchment area, UK.

**Participants:**

480 mothers involved in care proceedings with SUD between 2007 and 2019.

**Measurements:**

Substance use treatment records were linked to family court records. Kaplan Meier's time‐to‐event analysis was used to estimate the probability of returning to court and the recurrence rate. Hazard ratios (95% confidence intervals) were estimated to identify factors using Cox proportional regression analysis.

**Findings:**

Following the completion of the first care proceeding case, one‐quarter of the cohort returned to proceedings (*n* = 119). Of returning mothers, 58.0% returned with a new baby and 52.0% had not received SUD treatment during the first proceedings. The risk of returning was highest within five years and was positively associated with younger maternal age [adjusted hazard ratio (aHR) 0.26, 95% confidence interval (CI) = 0.11–0.61], multiple children in initial proceedings (aHR 2.07, 95% CI = 1.36–3.18) and not receiving SUD treatment during initial proceedings (aHR 0.42, 95% CI = 0.29–0.61). The number of contact events with SUD treatment was not statistically significantly associated with returning to proceedings.

**Conclusion:**

Among mothers receiving treatment for substance use disorder and involved in care proceedings in England, nearly one in four were likely to appear in a subsequent care proceeding case.

## INTRODUCTION

In recent years, the increase in public family law proceedings cases (‘care proceedings’) in England has highlighted the need to gain a better understanding of the factors associated with children entering care [1, 2, 3]. A significant issue in care proceedings is the number of mothers who return to proceedings after having children removed, only to have more children removed [4, 5, 6, 7]. One in four mothers who have been through care proceedings will return within 7 years [4], accounting for approximately 20.0% of care proceedings cases within England's public care system [8]. Similar trends are also observed in countries with comparable child protection systems, such as Australia [9, 10], Canada [11] and the United States (US) [12, 13]. Since the 2010s, England has introduced new initiatives to mitigate recurrent care proceedings [14, 15, 16]. However, despite progress, these services remain limited in number and scale, and face challenges, especially regarding sustainability and funding [17].

The majority of women who return to care proceedings have a history of substance use disorders (SUD) [17, 18]. SUD is prevalent in 50.0% to 80.0% of proceeding cases in Australia, England and the United States [19, 20, 21]. A similar proportion of mothers receiving treatment for SUD have experienced the loss of care for at least one child [22]. The removal of a child can worsen mental health difficulties and a resurgence of substance use, which impacts recovery [23]. Additionally, social and legal stigmatisation (sanctions on prohibiting family contact) along with welfare restrictions (housing, employment and welfare benefits), further complicate challenges after a child is removed [9, 23]. These consequences often cause mothers to fall through gaps between children's social care and SUD treatment services, re‐appearing only when pregnant again [23]. Neglecting the needs of mothers after child removal adversely affects mothers, children and wider society including extended family, agencies and public finances [24].

Our earlier work on mothers receiving treatment for SUD involved in care proceedings in England between 2007 and 2019 highlights concerns about the ability of the current model of care to identify and respond to maternal needs, with 82.1% (394/480) having their child placed in out‐of‐home care (i.e. care order, special guardianship order, child arrangements order, placement order or adoption) [25], which is far higher than the 50.0% prevalence for mothers without known SUD who enter care proceedings [26, 27, 28]. Empirical evidence on the factors that place mothers with SUD at greater risk for repeated care proceedings is very limited. Current evidence is restricted to US‐based samples, which show that mothers who entered SUD treatment sooner after their children were placed in out‐of‐home care [29], who stayed in treatment longer [30, 31, 32] and who completed at least one course of treatment were significantly more likely to be reunified with their children [33]. A better understanding of the role of SUD treatment utilisation and mothers reappearing in care proceedings is much needed. Without empirical evidence of the prevalence and characteristics of these cases, SUD treatment and social services will continue to face challenges in preventing their recurrence.

This study examined the recurrence of care proceedings among mothers attending treatment for SUD. We aimed to determine the prevalence and timing of returning to care proceedings as well as the profile of returning mothers. Additionally, we wanted to know if there was a difference in SUD treatment service use between mothers who returned to proceedings and those who did not. Specifically, we investigated whether service utilisation before and during the first care proceedings were associated with returning to court. We also explored the association between service utilisation and returning to court with the same child from first care proceedings and with a new child.

## METHODS

### Design and participants

We conducted a retrospective cohort study using linked Family Court and health administrative datasets from the United Kingdom (UK) [34]. Records of 480 mothers attending the South London and Maudsley (SLaM) National Health Service (NHS) Foundation Trust SUD services were extracted using the Clinical Records Interactive Search (CRIS) tool [35]. SLaM provides a range of SUD services (residential and community‐based programs) to a catchment area of approximately 1.36 million people within the London boroughs of Southwark, Lambeth, Lewisham and Croydon. Care proceedings data were accessed through the Children and Family Court Advisory and Support Service (Cafcass). Approval for this study was granted by the Cafcass Research Governance Committee and SLaM's CRIS oversight committee (ref: 21–103).

The sample included service users from SLaM substance use treatment services, identified as the mother of a child subject to care proceedings between April 2007 and March 2019. A full description of the linked cohort can be found in Pearson *et al*. [34], but in brief, a rule‐based approach with names, birth dates and postcode history, following the separation principle was used to link records [35]. This principle ensures privacy by preventing access to datasets that contain both personal identifiers and attribute data. Of 3226 women with a child involved in care proceedings in the SLaM area between 2007 and 2019, 2137 (66.2%) were linked to a SLaM patient record. Women in the linked cohort were generally younger at the birth of their first child than other women in England and most had just one child recorded in proceedings. Half had an infant (<12 months) subject to proceedings, aligning with previous research [36, 37]. A flow diagram of the study participants is presented in Figure S1.

### Outcome

Each mother's case index was defined as the first care proceeding case resulting in a final legal order. If multiple care proceedings were opened simultaneously, the younger child was chosen as the index case. We defined recurrent care proceedings cases as any mother involved in at least one new set of proceedings after the completion of the index case (no = 0, yes = 1). This includes any new application made during the observation period (2007–2019, inclusive) regardless of closure.

### Covariates

Further details on variables are included in Table S1. In brief, characteristics of the index care proceedings include the child's age (measured in weeks for children under 1 year old and in years for older children; also categorised as: under 4 weeks old = 1, 4 weeks to 11 weeks old = 2, 1–4 years old = 3, 5–9 years old = 4, 10 years old or above = 5), multiple cases opened simultaneously with the index case (no = 0, yes = 1) and the child's father involvement in the index case (no = 0, yes = 1). For mothers returning to care proceedings, we identified whether the subsequent case involved the same child (no = 0, yes = 1) or a new child including a newborn baby (defined as under 1 year old) (no = 0, yes = 1). We assessed the outcome of the index case to create a variable indicating out‐of‐home placement (no = 0, yes = 1), encompassing legal outcomes such as care order, special guardianship order, child arrangements order, placement order or adoption order [35].

Clinical and treatment characteristics include daily alcohol use (≥4 days in a week) (no = 0, yes = 1) and weekly use (at least once) of opiate (no = 0, yes = 1) or/and cocaine (no = 0, yes = 1) or/and crack use (no = 0, yes = 1), as recorded in the first treatment outcome profile (TOP) [38] assessment completed closest to the index case's open date. We measured substance use treatment service utilisation by extracting data on the frequency of contact events with services over eight quarters within a 24‐month window period for the index care proceedings. This included four quarters before and four quarters from the commencement date of the case. Each quarter represents 3 months, which reflects the clinical assessment window for SLaM SUD services. Any day a service user was an inpatient (for some or all of that day) or had contact in face‐to‐face, online and telephone clinical appointments such as outpatient visits and specialist team appointments (excluding home treatment visits) with SUD professionals was counted as one contact event. Contact events were recorded as a continuous variable rather than the absolute number of days to account for varying follow‐up periods. For cases with no recorded attendance (missing value), we used ‘no attendance’ and ‘discharge’ notes to determine if the patient was registered with any services.

Socio‐demographic characteristics of the mothers include age at the index case start, ethnicity (White/White British = 1, Other = 0), Index of Multiple Deprivations 2010 (IMD) quintiles (ranging from 1 most deprived to 5 least deprived) [39] and housing problems (acute/eviction) in the past 4 weeks, which were extracted from SLaM records [39].

### Statistical analyses

Data analyses were performed using Stata v18 [40]. Sample characteristics were described using counts and percentages or means and SDs as appropriate. To estimate the time of returning to care proceedings (from the end date of the index case to the start of the first repeat case) and the profile of returning mothers survival analysis was used. Kaplan Meier's time‐to‐event analysis accounted for censoring because of incomplete observation and variable follow‐up [41] and was used to compare groups via the log‐rank test. We calculated effect sizes of socio‐demographic, index case characteristics and clinical factors associated with recurrence to care proceedings as hazard ratios (HRs) using Cox proportional hazards regression models (95% CI) and assessed the proportional hazards assumption with Schoenfeld residual tests.

Further Cox regression procedures were used to model associations between the rate of contact events with SUD treatment services and returning cases. To assess the individual effect of each predictor (contact events during the 12 months before and from the start date of index care proceedings), we first ran separate models for each predictor, with the outcome variable being returned to care proceedings. We then constructed several models that controlled for sets of related covariates (out‐of‐placement for the index child and socio‐demographic factors) with the final model adjusting for all covariates examined to provide a fully adjusted estimate. Because of a high proportion of missing values (*n* = 182, 37.9%) and potential collinearity with housing problems, we excluded the IMD quintile from the adjusted models. Finally, Cox regression models were repeated for returning with a new child and returning with the same child from the index case as separate outcomes. Unadjusted and adjusted HRs (95% CI) were reported (further description of the models in Table S2). All models were based on complete case analysis, with no imputation performed for missing values. Models were interpreted using binary significance testing (*P* < 0.05).

The analysis plan was not pre‐registered, and results should be considered as exploratory. Researchers can access the linked data by contacting the CRIS administrator at cris.administrator@slam.nhs.uk. Access to the codes used in this study can be made by contacting the corresponding author.

## RESULTS

### Sample characteristics

Table 1 outlines the characteristics of the sample (*n* = 480). In summary, the average age of the mother at the start of the index care proceedings was 31.65 (SD = 7.13) years old and the majority were from a White background (*n* = 334, 70.2%) and lived in the two most deprived quintiles of IMD (*n* = 196, 66.7%). Housing problems were reported by 174 mothers (36.0%). The most reported substance of use was cocaine and/or crack cocaine (*n* = 170, 35.7%).

**TABLE 1:** Sample characteristics (*n* = 480).^a^

Characteristics	*n* (%)	Not returned	Returned
Socio‐demographic			
Mothers' age at the start of index case (mean, SD)	31.65 (7.13)	32.40 (3.34)	29.38 (5.88)
White/White British^b^	334 (70.2%)	257 (71.2%)	77 (64.7%)
Housing instability (acute/eviction)	174 (36.0%)	124 (34.3%)	50 (42.0%)
IMD quintile^b^			
Q1: Most deprived quintile	25 (8.5%)	21 (8.3%)	4 (5.2%)
Q2	171 (58.2%)	131 (52.9%)	40 (51.9%)
Q3	70 (23.8%)	58 (23.0%)	12 (15.6%)
Q4	26 (8.8%)	21(8.3%)	5(6.5%)
Q5: Least deprived quintile	6 (2.0%)	4 (1.6%)	2(2.6%)
Care proceedings characteristics (index case*)*			
Age of child index case (mean, SD)^c^	2.62 (1.40)	2.69 (1.49)	2.32 (1.24)
Under 4 weeks	170 (35.4%)	122 (33.8%)	48 (40.3%)
4–11 weeks	32 (6.7%)	22 (6.3%)	10 (8.4%)
1–4 y	139 (28.9%)	98 (27.1%)	41 (34.6%)
5–9 y	88 (18.3%)	71 (19.8%)	15(12.6%)
10–16 y	51 (10.6%)	43 (12.0%)	5(4.2%)
Multiple cases opened with index case	200 (41.7%)	143 (39.6%)	74(62.2%)
Father party	336 (70.0%)	250(69.2%)	86(72.3%)
Legal outcome of proceedings for index case			
Dismissed/order of not order	26 (5.4%)	20 (5.5%)	6(5.0%)
Family assistance/supervision order	60 (12.3%)	45(12.5%)	15(12.6%)
Special guardianship/child assistance order	152 (31.7%)	112(31.0%)	40(33.6%)
Care/secure accommodation order	113 (23.5%)	85(23.5%)	28(23.5%)
Placement/adoption	129 (32.3%)	98(27.1%)	31(26.9%)
Out‐of‐home placement	394 (82.1%)	295(81.7%)	99(83.2%)
Clinical characteristics			
Substance used in the past 28 days			
Alcohol consumed mostly every day of the week	103 (21.5%)	79(21.9%)	24(20.2%)
Opiates	133 (27.7%)	104(28.8%)	29(24.4%)
Cocaine/crack‐cocaine	170 (35.7%)	129(35.7%)	41(34.4%)
Received substance use treatment during index case	354 (73.5%)	267(73.9%)	57 (47.9%)
No. of contact events with addictions services during proceedings, index case (mean, SD)^d^	21.8 (21.92)	19.2(22.72)	22.30(20.56)
Recurrent care proceedings' cases	119 (24.8%)	–	–
Returning with index case child	50 (42.0%)	–	–
Returning with a new child	69 (58.0%)	–	–
Received substance use treatment during index case	58 (48.7%)	–	–
Received substance use treatment during returned case	86 (72.3%)	–	–

Abbreviations: IMD, Index of Multiple Deprivations.

^a^ A fuller table of cohort characteristics is available in both Data S4 and Canfiled et al. [25].

^b^ Missing values for White/White British = 4; IMD = 186 (% in these cases refers from completed cases).

^c^ Age of child recorded as 0 for those under 1 year old.

^d^ Contact events for the 24‐months window period.

Most index cases involved a child under the age of 5 years (*n* = 341, 71.0%) with a mean age of 2.62 (SD = 1.40). There were 200 cases where the mothers had two or more children at the start of the index care proceedings (41.7%), and in all these cases the index case child was part of a sibling group (two or more care proceeding cases opened at the same time). The child's father was involved in 70% of index cases (*n* = 336). Most mothers had the child from their index case placed in out‐of‐home care (*n* = 394, 82.1%) following care proceedings. Further details on cohort characteristics are available in Table S3 [25].

### Estimating the probability of reappearing in care proceedings, the time and profile of returning mothers

One‐quarter of the sample has reappeared in a subsequent set of care proceedings (*n* = 119), yielding an overall return rate of 50 per 1000 person‐years (95% CI = 40–60). Of the returned cases, 69 (58.0%) involved returning to care proceedings with another child. The majority of these cases involved a newborn baby (*n* = 62; 89.9%).

**FIGURE 1:**
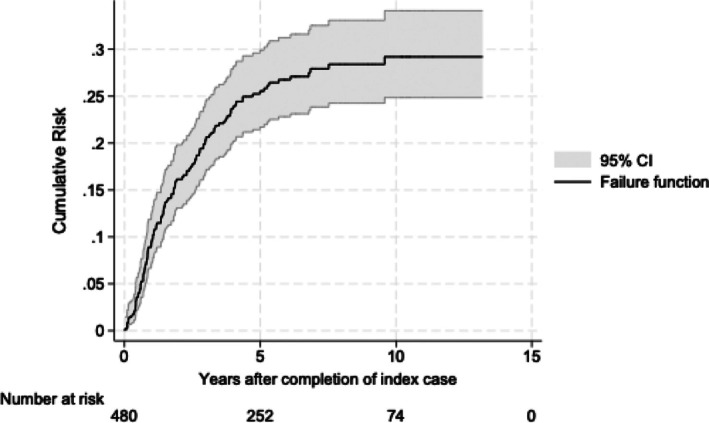
The cumulative risk of reappearing in care proceedings after the completion of the index case.

Figure 1 illustrates that the risk of a first repeat episode is greatest within the first 5 years after the completion of the index case. The cumulative risk of a mother entering the first repeated proceeding is 25.0% within 5 years, increasing to 27.0% within 7 years and 28.0% within 10 years.

The result of the log‐rank test indicates that there is evidence of a difference in survival curves according to the following factors: mother's age at the start of the index case [χ^2^ (3) = 12.5, *P* < 0.001], multiple cases open with the index case [χ^2^ (1) = 8.6, *P* = 0.040] and receiving treatment during index care proceeding case [χ^2^ (1) = 27.8, *P* < 0.001] (Figure S2). In the multivariable model, younger mothers (<25 years old) were more likely to return to proceedings than older mothers (>40 years old) [adjusted HR (aHR) = 0.26; 95% CI = 0.11–0.69, *P* = 0.039). Mothers with multiple cases at the index case were twice as likely to return than mothers with solo cases (aHR = 2.04; 95% CI = 1.36–3.18, *P* = 0.001). Mothers who received SUD treatment during the first care proceeding remained at a significantly lower rate of returning to proceedings compared to those who did not receive treatment (aHR = 0.42; 95% CI = 0.29–0.61, *P* < 0.001). Further descriptions of HRs for each variable included in the models (unadjusted and adjusted models) are reported in Table 2.

**TABLE 2:** Individual, clinical and index care proceeding characteristics associated with returning to care proceedings.

Characteristic	Unadjusted models		Adjusted models^a^	
	Hazard ratio (95% CI)	*P*‐value	Hazard ratio (95% CI)	*P*‐value
Socio‐demographic				
Mothers' age at start of proceedings for index case				
Up to 25 years old (reference group)				
26–32 years old	1.21 (0.98–1.24)	0.328	1.14 (0.91–1.20)	0.423
33–39 years old	1.10 (0.92–1.13)	0.301	1.07 (0.87–1.13)	0.350
40 and above	0.22 (0.09–0.56)	0.001	0.26 (0.11–0.61)	0.039
White/British White (no = 0)	0.80 (0.55–1.63)	0.244	0.83 (0.57–1.22)	0.314
Housing instability	1.06 (0.68–1.73)	0.347	1.04 (0.54–1.67)	0.487
IMD quintile^b^			**–**	***–***
Q1 (most deprived) (reference group)				
Q2	1.48 (0.53–4.12)	0.450		
Q3	1.14 (0.37–3.52)	0.823		
Q4	1.25 (0.33–4.65)	0.740		
Q5 (less deprived)	2.06 (0.38–11.27)	0.403		
Care proceedings				
Age of child index case^c^	0.91 (0.80–1.03)	0.141	0.83 (0.71–1.03)	0.057
Multiple cases opened with index case (no = 0)	1.69 (1.18–2.41)	0.004	2.07 (1.36–3.18)	0.001
Father party (no = 0)	1.04 (0.64–1.69)	0.324	1.16. (0.77–1.75)	0.499
Index case out‐of‐home placement (no = 0)	1.13 (0.65–1.96)	0.856	0.99 (0.61–1.63)	0.982
Clinical characteristics				
Received treatment during the index care proceeding case (no = 0)	0.40 (0.29–0.57)	<0.001	0.42 (0.29–0.61)	<0.001
Substance used in the past 28 days (no = 0)				
Alcohol consumed mostly every day of the week	0.89 (0.57–1.39)	0.613	0.80 (0.53–1.32)	0.437
Opiates	0.75 (0.50–1.14)	0.178	0.69 (0.44–1.17)	0.176
Cocaine/crack‐cocaine	1.01 (0.69–1.45)	0.986	1.01 (0.65–1.57)	0.956

^a^ Model adjusted for all variables (*n* = 476): Cox model χ^2^ 46.25 *P* < 0.001.

^b^ IMD Quintile not included in the adjusted model because of missing values.

^c^ Age of child index case measured as continuous (under 1 year old recorded as 0).

### Service utilisation and association with returning to care proceedings

**FIGURE 2:**
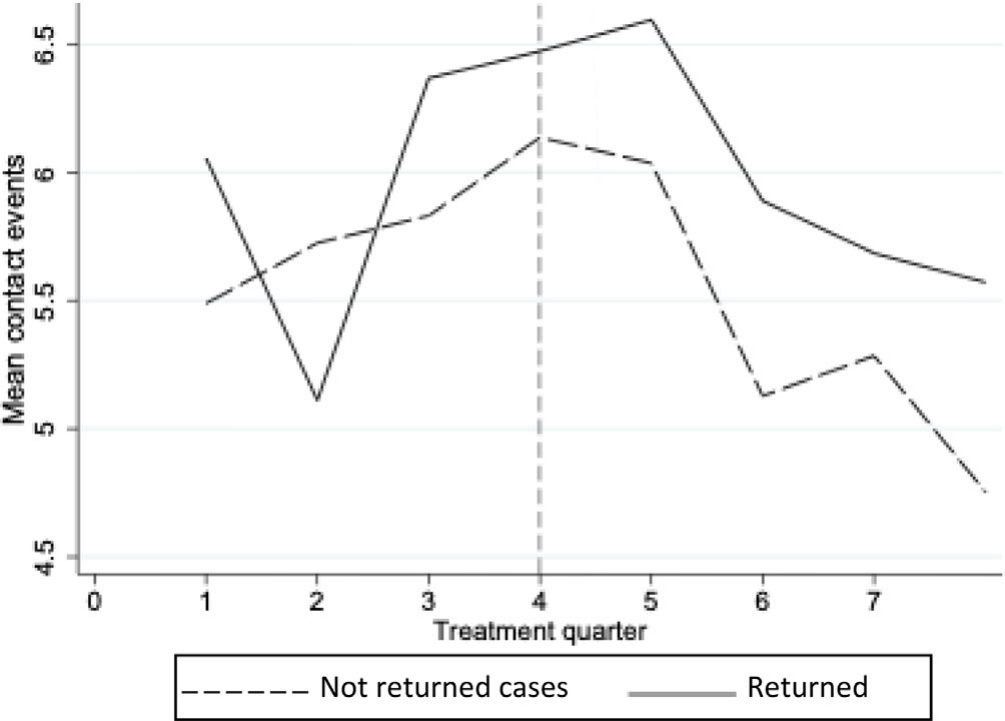
Mean contact events per substance use treatment quarter during proceedings for index case according to recurrence status (x axis = quarter 4 equivalent to the start date of index case's proceedings) Note: Contact events were measured as a continuous variable, representing the total number of interactions within each 3‐month treatment quarter (x‐axis). These included inpatient care, face‐to‐face, online and telephone clinical appointments, such as outpatient visits and specialist team consultations with substance use disorder (SUD) professionals, each counted as a single contact event. Returned cases are defined as any mother involved in at least one new set of proceedings after the completion of the index case (no = 0, yes = 1). This includes any new application made during the observation period (2007–2019, inclusive) regardless of closure.

As reported in Table 1, there were 126 cases (26.5%) that did not receive substance use treatment during the 24‐month observation period (defined as the 12 months before and 12 months after the initiation of proceedings for the index case). Figure 2 displays the mean contact events per treatment quarter. There was a total of 7809 substance use treatment contacts for the index cases during the 24 months, corresponding to an average of 21.8 (SD = 21.9) contact events.

Table 3 presents HRs and CIs for the association between contact events with SUD services and returning to care proceedings, which are presented according to any returning cases and for returning cases with the same child from the index case or a new one. There was no evidence that contact events with SUD services (either before or from the start date of the index care proceeding) predicted return to care proceedings. Likewise, no association was found in the event models examining return to care proceedings with the index case.

**TABLE 3:** Association between contact events with SUD services over 12 months before and from the starting date of the index's care proceedings case and returning to care proceeding according to any returning cases and returning pathway (same/new child from index case) (*n* = 354 cases, in treatment during proceedings).

	Returning to care proceedings		Returning with same child		Returning with new child	
	Hazard ratio (95% CI)	*P*‐value	Hazard ratio (95% CI)	*P*‐value	Hazard ratio (95% CI)	*P*‐value
Contact events 12 months before the starting date	0.91 (0.75–1.09)	0.304	1.17 (0.79–1.73)	0.417	1.09 (0.84–1.41)	0.489
Adjusted for 12 months after	0.90 (0.72–1.12)	0.356	1.05 (0.66–1.67)	0.826	1.14 (0.83–1.55)	0.400
Adjusted for index case out‐of‐home placement	0.90 (0.75–1.10)	0.300	1.13 (0.76–1.70)	0.538	1.07 (0.85–1.43)	0.441
Adjusted for socio‐demographics^a^	0.91 (0.73–1.14)	0.416	1.45 (0.59–1.53)	0.878	1.17 (0.85–1.62)	0.338
Fully adjusted	0.91 (0.73–1.14)	0.415	1.04 (0.65–1.65)	0.860	1.16 (0.83–1.60)	0.369
Contact events 12 months from the starting date	0.96 (0.82–1.25)	0.631	1.27 (0.84–1.92)	0.250	0.99 (0.82–1.21)	0.992
Adjusted for 12 months before	1.01 (0.82–1.25)	0.929	1.23 (0.75–2.03)	0.404	0.95 (0.74–1.20)	0.655
Adjusted for index case out‐of‐home placement	0.95 (0.79–1.14)	0.615	1.28 (0.84–1.94)	0.250	1.01 (0.83–1.22)	0.943
Adjusted for socio‐demographics^a^	0.94 (0.78–1.13)	0.524	1.38 (0.89–2.15)	0.141	1.01 (0.82–1.23)	0.954
Fully adjusted	0.99 (0.80–1.22)	0.922	1.43 (0.83–2.47)	0.198	0.96 (0.74–1.22)	0.727

Abbreviation: SUD, substance use disorder.

^a^ Adjusted models for mother's age at the start of the index case, White/Non‐White British, housing instability.

## DISCUSSION

In this retrospective cohort of 480 mothers receiving treatment for SUD who were involved in care proceedings, almost one in every four mothers was likely to reappear in a subsequent set of proceedings within 10 years after the completion of the first case. The risk of reappearing was greatest within the first 5 years and accounted for a rate of 50 per 1000 person‐years. These findings are consistent with previous studies that looked at reappearing cases in the general population of mothers in the English family courts [4, 23]. Consistent with previous studies were our findings that the risks of returning to care proceedings were higher for mothers who, in the first set of care proceedings, were younger and had multiple cases running simultaneously (more than one child in care proceedings) [4].

We report that while more mothers returned to court with a new child (58.0%), many mothers were returning to court with the same child from the index case (42.0%). In a previous study of the general population of mothers involved in care proceedings, the rates of returning to proceedings with the same child within the first 10 years of their first appearance were lower than those found in our cohort (25.8% in England and 15.7% in Wales) [7]. Although we do not have data on reasons for children entering or returning to care proceedings, over half of the mothers in our cohort (55.2%) had the index child moved into placement with a family relative or into foster care [25]. We also found that approximately half of the mothers who returned to proceedings had not received SUD treatment during the first set of care proceedings (48.7%), and over one‐quarter of mothers who returned to care proceedings had not received SUD treatment during the subsequent set of proceedings (27.2%). The risks of reappearing in care proceedings were higher for mothers who had not received SUD treatment during the first care proceeding case than for those who had received treatment. However, the absence of an association between the number of contact events that a mother had with SUD treatment services during the first care proceedings and rates of reappearance in care proceedings limits our interpretation of the role of SUD treatment services in preventing reappearance cases. For instance, it is unclear whether low or high engagement with services might serve as a proxy for addiction severity, potentially increasing the risks of returning to care proceedings. The lack of comparable data on this topic means that we do not know whether addressing SUD alone is sufficient to break the cycle of repeat care proceedings. We do know, however, that as well as SUD, mothers in care proceedings experience multiple disadvantages including domestic violence, early childhood adversities, mental health comorbidities, poverty, housing instability and criminal justice involvement [42, 43]. Working with mothers with SUD involved in care proceedings means recognising that substance use is not an isolated problem, and that the causes and consequences of substance use are interconnected with a range of social and psychological factors, which are often cumulative and reinforce one another [44, 45, 46].

### Implications

Existing evidence makes a clear case for better provision of SUD treatment services for mothers including those involved in care proceedings [42, 47, 48]. Our findings further recognise that many mothers with SUD involved in care proceedings do not receive the necessary support during times of crisis. Findings highlight that a lack of sufficient engagement with SUD services—whether through individual maternal difficulties to accept support or the limitations in service provision to deliver adequate support—not only increases the likelihood of mothers having their child placed into out‐of‐home care [25], but also raises the probability of them returning to future proceedings. Social services need to be equipped with the necessary resources (e.g. standardised assessment tools, stigma reduction training) and skills (e.g. recognise when substance use and/or other mental health issues become a safeguarding concern) to effectively engage in discussions with mothers about substance use and their need for support.

Our findings also support calls from England and Wales for services to act faster when mothers have their children removed from their care [2, 7, 18]. Timely access to SUD treatment for these women is likely to improve treatment outcomes including retention and engagement [49]. Treatment progression is an important factor for reunification [47]. Higher rates of reunification are more likely to occur when the multiple needs of the mothers are addressed while in treatment. This includes improving social determinants of health such as social networks and support, education, employment and economic issues [48]. When reunification is not achievable, efforts in SUD treatment continue to impact the mother and potential future children positively [50, 51]. Further engagement with SUD services should equip mothers with better internal resources to rebuild and sustain a healthier relationship with their children. Investment in alliances between SUD treatment services and agencies should be a key priority for policymakers and commissioners. This could involve the implementation of statutory frameworks for post‐proceeding cases shared across agencies, which support mothers in overcoming the multiple formal consequences of having a child removed (i.e. reductions in welfare entitlements, legal stigmatisation) as well as informal (i.e. grief responses, social stigma).

Despite the best efforts of mothers and professionals, treating SUD is a fluctuating and lengthy process. Sustaining positive change can be challenging for mothers who are reunited with their children after the proceedings end. Evidence shows that the risk period for recurring risky parenting behaviours (domestic abuse, mental health difficulties and substance misuse) is typically within the first 2 years post‐proceedings [52]. The immediate post‐proceedings phase is when mothers are most vulnerable and in greatest need of support. All services involved (e.g. family court, social services and substance use treatment services) should collaboratively establish plans for frequent contact and oversight. Supporting permanency is an ongoing process requiring tailored services for mothers and their children at different levels and time points.

### Strengths and limitations

A key strength of this study is the use of linked administrative data from public family law and mental health service records. Analysing routinely collected data provides unique insights into individual health service patterns, overcoming the several challenges that exist in engaging mothers with substance use problems in research (i.e. stigma, privacy concerns and time constraints) [53]. This approach also reduces selection bias, self‐report bias and attrition, which are common in longitudinal studies.

A limitation of our study is that the probability of recurrence we presented is likely to be underestimated for key reasons. First, mothers' index set of care proceedings may not have been their first set of care proceedings as we only had data on proceedings from April 2007. Second, the duration of the data analysed may be insufficient to explore future child removal for mothers who had their index case in the most recent years. Third, our study focused solely on formal legal proceedings. In England, children can be placed in out‐of‐home care voluntarily (care arrangement under Section 20 of the 1989 Children Act) or compulsorily (court order under Section 31 of the 1989 Children Act)(54). Therefore, women who did not return to proceedings may still have experienced out‐of‐home care for another child. Our findings may be limited by confounding by indication, as mothers in SUD treatment might have characteristics linked to a higher risk of returning to care proceedings, making it difficult to determine if returns are because of service utilisation or other risk factors (e.g. severity of the addiction). Changes in SUD service provision to non‐SLaM providers within the SLaM catchment area over the observation window mean that rates of service utilisation are also likely to be underestimated. In addition, we have no information about migration out of the SLaM catchment area, which could lead to an underestimation of service use activity. A further limitation is a potential bias in the CRIS‐Cafcass linkage process, where quality issues (e.g. missing birth dates) and healthcare access disparities may have affected the linkage rate, possibly underestimating the true prevalence of women in care proceedings and those who return. Finally, because our cohort includes only mothers in SUD treatment, we lack data on mothers with substance use problems who never accessed SUD services and their likelihood of returning to care proceedings.

## CONCLUSIONS

Our study shows that many mothers attending SUD treatment who are involved in care proceedings do return to court in a new set of proceedings. It highlights that these mothers often, and repeatedly, do not receive the necessary support during times of crisis. Although our findings suggest that SUD treatment might help prevent repeated family court involvement, the exact mechanism remains unclear, as simply increasing opportunities for contact with SUD treatment services does not reduce reappearance rates. Our study supports existing calls for timely support for mothers after the completion of a care proceeding, regardless of its outcome (e.g. permanency or out‐of‐home placement). All services involved (family court, social services and SUD treatment) must work collaboratively to address service mechanisms that currently hinder mothers' ability to avoid repeated family court involvement.

## AUTHOR CONTRIBUTIONS

**Martha Canfield:** Conceptualization (equal); data curation (lead); formal analysis (lead); funding acquisition (equal); methodology (equal); project administration (lead); writing—original draft (lead); writing—review and editing (lead). **Gail Gilchrist:** Conceptualization (equal); funding acquisition (equal); project administration (equal); supervision (lead); writing—original draft (equal). **Johnny Downs:** Conceptualization (equal); funding acquisition (equal); methodology (equal); writing—original draft (equal). **Sam Norton:** Conceptualization (equal); formal analysis (equal); funding acquisition (equal); methodology (equal); project administration (equal); writing—original draft (equal).

## DECLARATION OF INTERESTS

None.

## Supporting information


**Figure S1.** Study participation flow diagram.
**Table S1.** Description of study variables.
**Table S2.** Further description of variables included in the Cox Regression models exploring the association between contact events with SUD services over 12 months before and from the starting date of the index's care proceedings case and returning to care proceeding according to any returning cases and returning pathway.
**Table S3.** Further Characteristics of the study cohort (N = 480), first reported in Canfield et al. (2023).
**Figure S2.** Cumulative risk of reappearing to care proceedings according to mother's age, multiple cases opened within the index case, and receiving treatment for SUD during proceedings for the index case.

## Data Availability

Data is available through a data request application to the Clinical Records Interactive Search (CRIS) and Children and Family Court Advisory and Support Service (Cafcass).
