# Supplementary material for: Recurrent care proceedings and use of services for substance use disorder: A retrospective linked data cohort study of mothers in South London
Source: Addiction. 2025 Aug 27;121(1):163–72. doi: 10.1111/add.70179 (PMC12710810; doi:10.1111/add.70179)
Supplement: Supplementary file 1 — Figure S1. Study participation flow diagram. Table S1. Description of study variables. Table S2. Further description of variables included in the Cox Regression models exploring the association between contact events with SUD services over 12 months before and from the starting date of the index's care proceedings case and returning to care proceeding according to any returning cases and returning pathway. Table S3. Further Characteristics of the study cohort (N = 480), first reported in Canfield et al. (2023). Figure S2. Cumulative risk of reappearing to care proceedings according to mother's age, multiple cases opened within the index case, and receiving treatment for SUD during proceedings for the index case. [file ADD-121-163-s001.docx]

**Figure S1.** Study participation flow diagram

Women involved in care proceedings in South London between April 2007 and March 2019 in Cafcass

(n= 5463)

Cafcass Linkage with CRIS

Women involved in care proceedings in South London between 2007 and March 2019 who did not link to a SLaM mental health service user record

(n=2620)

Women involved in care proceedings in South London between 2007 and March 2019 who linked to a SLaM mental health service user record

(n=2806)

Women identified as attending SUD treatment services

(n=480)

Women identified with at least one new set of proceedings after the completion of the index case during the observation period (2007–2019).

(n=119)

| **Variable** | **Value** | **Source** | **Description of how was measured and/or defined** |
| --- | --- | --- | --- |
| Mothers’ age at the start of index case | Continues | Family Court Dataset | In years |
| White / White British | No = 0; Yes = 1 | SLaM dataset | Non White/British white include Black/Black British, Asian/British Asian, Mixed Heritage and Other^1^ |
| Housing instability (acute/eviction) | No = 0; Yes = 1 | SLaM dataset | Assessed by the  Treatment Outcome Patients Form (TOP)^2^ completed nearly to the start of care proceedings |
| IMD Quintile | Q1 (most deprived) = 1;  Q2 = 2; Q3 = 3; Q4 = 4;  Q5 (least deprived) = 5 | SLaM dataset | A measure of relative deprivation in the UK  to assess deprivation across seven domains: income, employment, education, health, crime, housing, and the living environment.^3^ |
| Age of child index case | Under 4 weeks = 1; 4 weeks – 1year = 2; 1 – 4years old = 3; 5 – 9 years old = 4; 10 – 16 years old = 5 | Family Court Dataset | Measured in weeks if under one year old and in years otherwise |
| Multiple cases opened with index case | No = 0; Yes = 1 | Family Court Dataset | Two or more cases opened at the same time of the index case |
| Father party | No = 0; Yes = 1 | Family Court Dataset | Child’s father is party to the proceeding |
| Legal outcome of proceedings for index case | No = 0; Yes = 1 | Family Court Dataset | Data was extracted using the framework applied in previous research^4,5^: Dismissed/ Order of Not Order; Family assistance / Supervision Order; Care / Secure Accommodation Order; Placement / Adoption |
| Out-of-home placement | No = 0; Yes = 1 | Family Court Dataset | Parental responsibility curtailed or terminated, which include Care Order, Special Guardianship Order, Child Arrangements Order, and Placement Order or Adoption Order |
| Substance used in the past 28 days |  | SLaM dataset | Assessed by the  Treatment Outcome Patients Form (TOP)^2^ completed nearly to the start of care proceedings |
| Alcohol consumed mostly every day of the week | No = 0; Yes = 1 | SLaM dataset | Drinking four or more days in a week |
| Opiates | No = 0; Yes = 1 | SLaM dataset | Consumed at least once during the week |
| Cocaine/ crack-cocaine | No = 0; Yes = 1 | SLaM dataset | Consumed at least once during the week |
| Received substance use treatment during index case | No = 0; Yes = 1 | SLaM dataset & Family Court Dataset | Cases started before or during the start date of the care proceeding where treatment was ongoing during the time of care proceedings |

**Table S1.** Description of study variables

| **Variable** | **Value** | **Source** | **Description of how was measured and/or defined** |
| --- | --- | --- | --- |
| Number of contact events with addictions services during proceedings, index case | Continues | SLaM dataset & Family Court Dataset | Measured during a 24-month window period for the index care proceedings (8 quarters, each 3 months). This includes: 4 quarters before, 4 quarters after index case started. Contact events was defined as any day with: Inpatient stay (partial or full day); Face-to-face, online, or telephone clinical appointments; Outpatient visits and specialist team appointments. **Exclusions:** Home treatment visits. |
| Recurrent care proceedings’ cases | No = 0; Yes = 1 | Family Court Dataset | At least one new set of proceedings after the completion of the index case, including any new application made during the observation period (2007–2019, inclusive) regardless of closure |
| Returning with index case child | No = 0; Yes = 1 | Family Court Dataset | If the returning case refers to the same child from index case |
| Returning with a new child | No = 0; Yes = 1 | Family Court Dataset | If the returning case refers to a different child from index case including a newborn child (less than a year old) |
| Received substance use treatment during returned case | No = 0; Yes = 1 | Family Court Dataset | Cases started before or during the start date of the returning care proceeding case where treatment was ongoing during the time of care proceedings |

**References**

1. NHS England (2021) ethnic category. Available from https://datadictionary.nhs.uk/data_elements/ethnic category.html
2. Marsden J, Farrell M, Bradbury C, Dale-Perera A, Eastwood B., Roxburgh M., et al. Development of the treatment outcomes profile. *Addiction*. 2008; 103, 1450–1460
3. Ministry of Housing Communities & Local Government. English indices of deprivation. 2010 [cited 2019 Oct 21]. Available from: https://www.gov.uk/government/statistics/ english-indices-of-deprivation-2010
4. Bedston, S. J., R. J. Pearson, Matthew A. Jay, Karen Broadhurst, Ruth Gilbert, and Linda Wijlaars. "Data resource: Children and Family Court Advisory and Support Service (Cafcass) public family law administrative records in England." *Int J Popul Data Sci*. 2020; 5: 1159.
5. Pearson RJ, Jewell A, Wijlaars L, Bedston S, Finch E, Broadhurst K, Gilbert R. Linking data on women in public family law court proceedings concerning their children to mental health service records in South London. *Int J Popul Data Sci. 2021; 6*: 1385.

**Table S2**. Further description of variables included in the Cox Regression models exploring the association between contact events with SUD services over 12 months before and from the starting date of the index’s care proceedings case and returning to care proceeding according to any returning cases and returning pathway

| **Models** | **Variables** |
| --- | --- |
| Outcomes | Returned to care proceedings /returning with index case child, returning with a new child |
| Predictors | Contact events 12 months before the starting date of index case / Contact events 12 months after the starting date of index case |
| Adjusted for 12months after/before | Contact events 12 months after/before the starting date of index case |
| Adjusted for index case out-of-home placement | Out-of-home placement |
| Adjusted for socio demographics | Mother’s age at the start of the index case, ethnicity, housing instability |
| Fully adjusted | Contact events 12 months after/before the starting date of index case, out-of-home placement, mother’s age at the start of the index case, ethnicity, housing instability |

**Table S3.** Further Characteristics of the study cohort (N=480), first reported in Canfield et al. (2023)

| **Characteristics** | **N(%)** |
| --- | --- |
| Ethnicity |  |
| White / White British | 334 (75.6%) |
| Black / Black British | 89 (18.7%) |
| Asian or Asian British | - |
| Mixed Heritage | 39 (8.2%) |
| Other | 14 (2.9%) |
| Pregnant at time of assessment | 86 (25.5%) |
| Legal outcome of proceedings for index case |  |
| Dismissed/ Order of Not Order | 26 (5.4%) |
| Family assistance / Supervision Order | 60 (12.3%) |
| Special Guardianship / Child Assistance Order | 152 (31.7%) |
| Care / Secure Accommodation Order | 113 (23.5%) |
| Placement / Adoption | 129 (32.3%) |
| Length of time of proceedings |  |
| Up to 26 weeks | 131 (27.1%) |
| Above 26 weeks | 349 (72.7%) |
| Median average number of weeks (25%-75% quintiles) | 37.1 (25.3; 52.7) |
| Fiscal Year of starting index case |  |
| 2007/2008 | 42 (8.7%) |
| 2008/2009 | 59 (12.3%) |
| 2009/2010 | 49 (10.2%) |
| 2010/2011 | 41 (8.5%) |
| 2011/2012 | 49 (10.2%) |
| 2012/2013 | 78 (16.2%) |
| 2013/2014 | 36 (7.5%) |
| 2014/2015 | 37 (7.7%) |
| 2015/2016 | 22 (4.6%) |
| 2016/2017 | 36 (7.5%) |
| 2017/2018 | 23 (4.8%) |
| 2018/2019 | 8 (1.7%) |
| 2007/2008 to 2013/2014 | 354 (73.7%) |
| 2014/2015 to 2019/2020 | 126 (26.2%) |
| Treatment Status |  |
| Median average length of time in treatment in weeks (quintiles) | 148.5 (46.2; 320.3) |
| Entered treatment before index case started | 237 (40.4%) |
| Median average length of time in treatment before case started in weeks (25%-75% quintiles) | 62 (18.4, 145.0) |

**Reference**

Canfield M, Norton S, Downs J, Wijlaars LPMM, Gilchrist G. Risk factors for involvement in care proceedings for mothers receiving treatment for substance use: a cohort study using linked and administrative data in South London. Child Youth Serv Rev. 2023; 155: 107180

**Figure S2.** Cumulative risk of reappearing to care proceedings according to mother’s age, multiple cases opened within the index case, and receiving treatment for SUD during proceedings for the index case
